# Lab-on-chip (LoC) application for quality sperm selection: An undelivered promise?

**DOI:** 10.12688/openreseurope.16671.1

**Published:** 2023-10-30

**Authors:** Shiva K Shukla, Pierre Gaudriault, Antoni Corbera

**Affiliations:** 1 Research and Development Unit, Beez Biotech SAS, RENNES, Ille-et-Villain, 35000, France; 2 Research and Development Unit, Cherry Biotech SAS, Paris, 93100, France

**Keywords:** Microfluidics, sperm selection, rheotaxis, thermotaxis, chemotaxis

## Abstract

Quality sperm selection is essential to ensure the effectiveness of assisted reproductive techniques (ART). However, the methods employed for sperm selection in ART often yield suboptimal outcomes, contributing to lower success rates. In recent years, microfluidic devices have emerged as a promising avenue for investigating the natural swimming behavior of spermatozoa and developing innovative approaches for quality sperm selection. Despite their potential, the commercial translation of microfluidic-based technologies has remained limited.

This comprehensive review aims to critically evaluate the inherent potential of lab-on-chip technology in unraveling sophisticated mechanisms encompassing rheotaxis, thermotaxis, and chemotaxis. By reviewing the current state-of-the-art associated with microfluidic engineering and the swimming of spermatozoa, the goal is to shed light on the multifaceted factors that have impeded the broader commercialization of these cutting-edge technologies and recommend a commercial that can surmount the prevailing constraints. Furthermore, this scholarly exploration seeks to enlighten and actively engage reproductive clinicians in the profound potential and implications of microfluidic methodologies within the context of human infertility.

## Introduction

Quality sperm separation is essential in accomplishing the practices associated with artificial reproductive techniques (ART). In order to prevail over the health issues related to human infertility, ART practices include intracytoplasmic sperm injection (ICSI), intrauterine insemination (IUI), and *in vitro* fertilization (IVF) treatments.

Since the last decade, the market for ART has been growing unceasingly ( Aderaldo *et al.*, 2023). Increasing infertility rates, technological advancements, government policies, and the involvement of private actors appeared as chief market drivers ( Patrizio *et al.*, 2022). On the contrary, the higher cost and failure rates emerged as potential marketing constraints. According to the “European Society of Human Reproduction and Embryology (ESHRE)”, the average possibility of pregnancy and delivery per embryo transfer is 37% and 21%, respectively ( De Geyter *et al.*, 2018). Substandard *in vitro* conditions, quality of male/female gametes, and damages related to embryos are the potential factors leading to the failures of ART. The separation of high-quality spermatozoa from semen samples is a significant step, and the efficacy of ART is majorly correlated with it ( Oseguera-López *et al.*, 2019; Pérez-Cerezales *et al.*, 2018; Sakkas *et al.*, 2015).

The “World Health Organization (WHO)” protocol for sperm preparation involves standard centrifugation-based practices, including sperm wash, density gradient centrifugation (DGC)”, and sperm swim-up ( World Health Organization, 2021), which causes DNA fragmentation in spermatozoa ( Alvarez *et al.*, 1993). Fernandez-Gonzalez *et al.,* validated the side effects of utilizing damaged male gametes in a mouse model where the substandard cells were proficient in fertilizing oocyte; however, it leads to an alteration in gene expression and promotes a defective fetal/placental development ( Fernández-Gonzalez *et al.*, 2008).

Regardless of these technological drawbacks, the DGC and swim-up have remained the most practiced protocols for quality sperm separation over the past 40 years ( Rappa *et al.*, 2016). Consequently, there is an enormous possibility of technological upgradation, which can assist in advancing ART outcomes. Sakkas *et al.* recommended connecting the missing elements in quality sperm cell selection and encouraged to replicate the natural selection process ( Sakkas *et al.*, 2015). Three main mechanisms, explicitly rheotaxis, chemotaxis, and thermotaxis, are known to direct sperm cells toward oocytes. Rheotaxis justifies the swimming and rolling of sperm cells against the flow direction. Thermotaxis —migration of sperm cells induced by temperature gradient— is theorized for directing the swimming of sperm cells through the follicular tube. Chemotaxis evokes the redirection of sperm cells towards oocytes and triggers sperm cell accumulation ( Giojalas & Guidobaldi, 2020; Pérez-Cerezales *et al.*, 2018; Suarez, 2016; Suarez & Pacey, 2006). Emphatically, the female reproductive tract facilitates the microenvironment, enabling the selection of high-quality spermatozoa for *in vivo* conception.

The *in vivo* mechanisms of spermatozoa swimming are complex phenomena; henceforth, translating these mechanisms to *in vitro* settings is not straightforward. Nonetheless, lab-on-a-chip (LoC) researchers have successfully leveraged the advantages of microfluidic technology and established the proof-of-concept (PoC) in relation to the selection of high-quality sperm cells.

Microfluidic engineering involves the manipulation of small volumes ranging from nanoliters (nL to milliliters (mL). The small-volume scale and sub-millimeter channel dimension comprise a unique feature: the fluid motion in parallel streams, known as laminar flow, where the ratio of inertial and viscous forces is meager. This dimensionless ratio is known as *Reynolds (Re)* number, and it computes the predisposition of the fluid motion to develop turbulence ( Bruus, 2008). The laminar flow through the microchannel enables a high degree of control, and this characteristic brings numerous advantages compared to conventional laboratory practices. The microfluidic system utilizes low sample and reagent volumes, which reduces the operational cost and improves the sensitivity and rapidness of the associated biological protocol. Microfluidic offers parallel processing, which results in high yields; moreover, technology can be integrated with external manipulation, including acoustics ( Clark *et al.*, 2019), optics ( Schiffer *et al.*, 2020), magnetic ( Xu *et al.*, 2020), electric ( Chen *et al.*, 2013a; De Wagenaar *et al.*, 2016a) for cell analysis.

In application to sperm cell separation, microfluidic-based approaches are capable of producing biomimetic environments, primarily associated with temperature and chemical changes, to examine the navigation of sperm cells under conditions that resemble the *in-vivo* landscape. Additionally, the technology can replicate the anatomical confinement of the female reproductive tract, allowing to unravel the impact of fluidic shear and geometrical constraints on swimming of sperm cells. However, the commercial translation of LoC-based devices —particularly for the mentioned field— is inferior. Furthermore, post-commercialization of a few microfluidic devices, the clinical outcomes of the ART practices remain unchanged.

This review endeavors to project an extensive overview of microfluidic devices, unravelling the natural swimming mechanisms of sperm cells and propose potential advancements for their incorporation in fertility treatments, specifically for quality sperm selection. We have created a reference database through Pubmed® and reviewed the last ten years of research articles attributed to keywords including rheotaxis, chemotaxis, thermotaxis, and sperm separation concerning microfluidic technology. This study sheds light on the primary factors influencing the low commercial translation of microfluidic-based technologies for sperm separation despite their demonstrated capabilities in exploring natural swimming mechanisms. Subsequently, we propose a pathway for commercializing microfluidic technologies and translating them from the laboratory into the clinical environment.

## Microfluidics: a potential toolbox to unravel sperm cell dynamics

### Rheotaxis

Sperm cells entering the vagina have to endure an acidic microenvironment, as the pH level of vaginal discharge typically falls within the range of 4 to 4.5 ( Prins, 2010). Subsequently, cervical mucus promotes rheotaxis, resulting in the separation of non-spermatozoa cells, pathogens, and non-motile sperm cells. The swimming of sperm cells through the cervical canal is not entirely understood. However, the animal model suggests that the microgrooves expedite the upstream swimming of sperm cells toward the uterus ( Suarez, 2016).

The *in vivo* locomotion of the sperm cells occurs at a low *Reynolds (Re)* number, where they create fluid flow patterns by repelling the adjacent fluid at the back and front while propelling themselves forward through their beating flagellum. The migration of sperm cells through a microchannel is a close replication of *in vivo* conditions. A microfluidic device featuring a rectangular or circular microchannel connected to a fluid inlet and an outlet can exhibit rheotaxis. The flow control along the microchannel is established through the “active” or “passive” methods. The “active” approach enables controlling of fluid flow *via* an external fluid-injector unit like a syringe, peristaltic, or pressure pump ( Bukatin *et al.*, 2015; Kantsler *et al.*, 2014; Miki & Clapham, 2013; Nishina *et al.*, 2019; Romero-Aguirregomezcorta *et al.*, 2018; Schiffer *et al.*, 2020; Tung *et al.*, 2014; You *et al.*, 2019a; Zaferani *et al.*, 2019; Zhang *et al.*, 2016). Conversely, the “passive” method involves the flow induction through the hydrostatic pressure difference between the inlet-outlet of the connected microchannels ( Abdel-Ghani *et al.*, 2020; El-sherry *et al.*, 2017; El-Sherry *et al.*, 2014; EL-sherry *et al.*, 2020; Hyakutake *et al.*, 2021). The active flow system facilitates a high degree of flow precision but augments the expense and complexity of the experimental setup. On the contrary, the passive flow unit offers a relatively inexpensive and standalone system; however, the flow precision can be compromised.

The laminar fluid streams across the microchannel exhibit a parabolic profile, and the transportation of spermatozoa in the upstream direction abides by a helical trajectory. The spiral pattern is the most apparent swimming pattern for sperm cells, enabling them to cover a larger oviductal region. The shear flow through the microchannel complements the apparent and curvilinear velocities, which augment the progressiveness and chirality of swimming sperm ( Kantsler *et al.*, 2014). Reviewed studies have demonstrated that sperm cells exhibit rheotaxis in human and bovine models, with observed velocity ranges of 20 µm/s to 150 µm/s ( Kantsler *et al.*, 2014; Nishina *et al.*, 2019). On the other hand, increased viscosity (1 mPa s to 20 mPa s) deaccelerates progressiveness and chirality ( Kantsler *et al.*, 2014; Zaferani *et al.*, 2021a).

**Figure 1.**
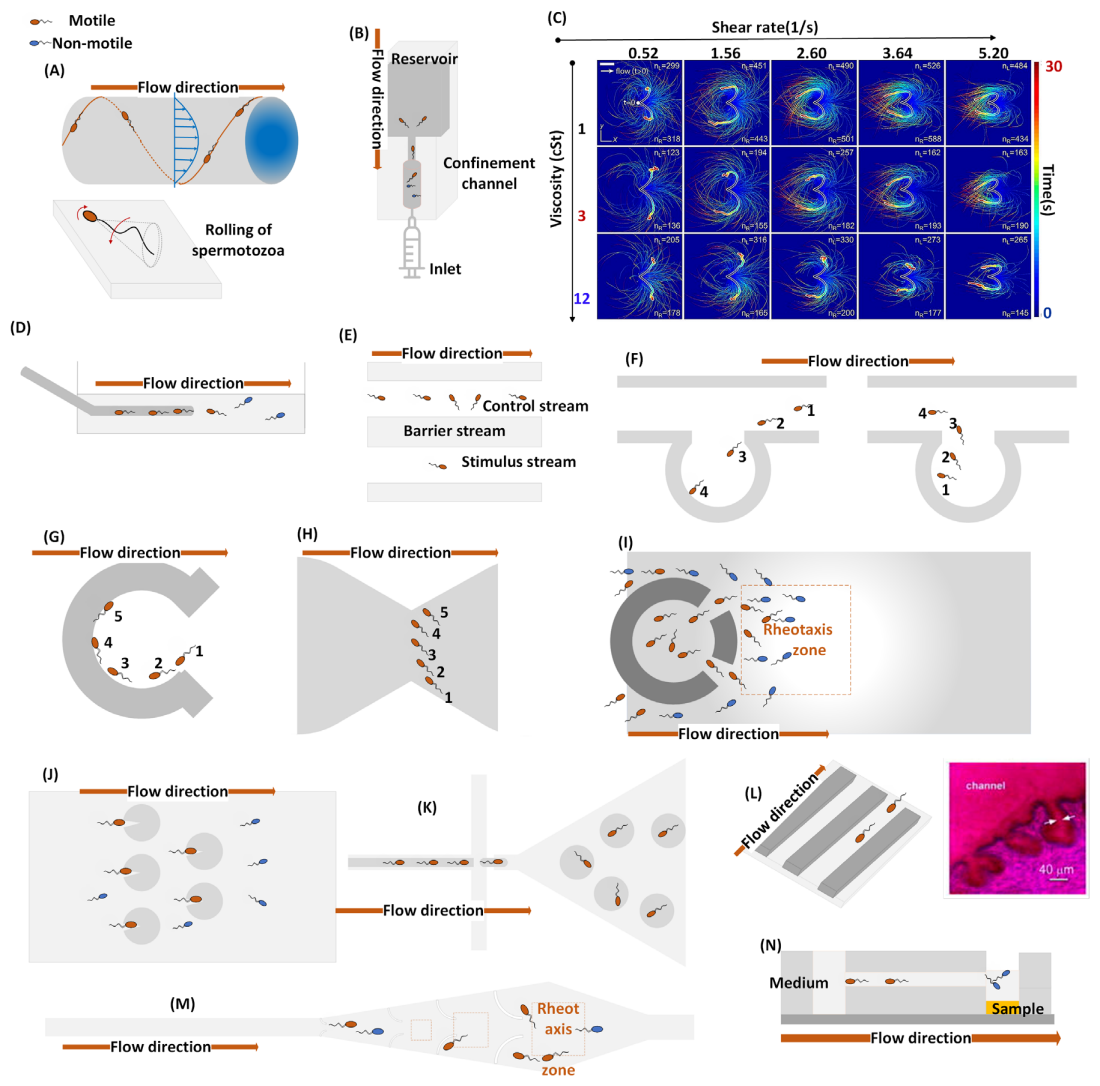
Microfluidic models for the demonstration of sperm rheotaxis. Figure ( **A**) illustrates the investigation conducted by ( Kantsler *et al.*, 2014) on rheotaxis (at 20-60 µm/s) induced chirality in a circular microchannel (0.34mm × 3mm) for the bovine and human model. Figure ( **B**) depicts the experimental setup of ( Nishina *et al*., 2019) for studying the antigravity and confined swimming of human sperm using a circular microchannel (50, 70, 100 µm). Figure ( **C**) is copied from ( Bukatin *et al.*, 2015), demonstrates the reorientation of bovine sperm cells in response to shear rates (0.52 - 5.2 s ^-1^) in a simple microchannel (W×H: 0.29mm ×3mm). Figure ( **D**) replicates the rheotaxis setup (50µm/s and 90 µm/s) for mouse and human cells using a glass dish and an 80µm and 125 µm capillaries, respectively ( Miki & Clapham, 2013; Zhang *et al.*, 2016). Figure ( **E**) represents an optical trapping setup integrated with microfluidic capillaries (H×W: 0.4µm×0.33µm) for CatSper induced reorientation study in human sperm ( Schiffer *et al.*, 2020). Figure ( **F**) illustrates the rheotactic behaviors (33–101 µm/s) of bovine sperm cells through microchannels (W×H: 200µm × 20µm and 50µm × 20µm) with side pouches ( El-Sherry *et al*., 2014). Figure ( **G**) explains rheotaxis spermatozoa through curved wall coral design (D: 500µm, H: 25µm) in viscous flow (1.ml/h) ( Zaferani *et al.*, 2018; Zaferani *et al.*, 2021a). A similar approach was utilized for cell entrapment in ( Yaghoobi *et al.*, 2022). Figure ( **H**) demonstrates the butterfly-like upstream swimming (0-2ml/h) of human/bovine sperm in narrowed microchannels (W×H:40×30µm) ( Zaferani *et al.*, 2019). Figure ( **I**) reproduced from ( Zaferani *et al*., 2018) device comprises the corral design, which facilitates the rheotaxis zone (40–90 µm/s) for bovine/human and the entrapment of the motile cell. Figure ( **J**) demonstrates the rheotaxis-based human sperm cells trapping micro-system (0.5ml/min) with 32µm channel depth and 2µm depth for a circular array ( You *et al.*, 2019b). Figure ( **K**) mimics the complex topography of epithelial tissue by trapping the bovine sperm cells into droplets sized 90–120µm ( Raveshi *et al.*, 2021). Figure ( **L**) represents microgrooves (8mm × 20µm × 20µm) integrated into microchannels (4cm × 300µm × 120µm) to replicate the bovine sperm rheotaxis (1-3µl/min) with surface inhomogeneities ( Tung *et al.*, 2014; Tung *et al.*, 2015). Figure ( **M**) illustrates the capturing of motile human sperm cells using a microchannel (1.5mm × 450µm ×100µm) containing micro-pockets with curvature of 150–300 µm ( Sarbandi *et al*., 2021). Figure ( **N**) shows a passive-flow rheotaxis (60µm/s) using a microchannel (5mm × 200 µm × 20 µm) to unravel bovine sperm-wall interaction ( Hyakutake *et al.*, 2021).

Additionally, sperm cells showed a high propensity to move along the wall or micro-pockets ( El-Sherry *et al.*, 2014; Heydari *et al.*, 2023; Hyakutake *et al.*, 2021; Nosrati *et al.*, 2016; Sarbandi *et al.*, 2021; Yaghoobi *et al.*, 2022; Zaferani *et al.*, 2018). The conical envelope of the flagella beating promotes the wall and spermatozoa interaction. Adjacent to the wall, the magnitude of the flow streams is minimal due to no-slip boundary conditions, where the sperm head is hardly affected by the fluid streams, and the flagellum encounters a greater force. The studies claim that the confinement provokes rheotaxis and helical turns in the human and bovine models ( El-Sherry *et al.*, 2014; Nishina *et al.*, 2019). Hence, the interaction between the reproductive tract and spermatozoa appeared as the principal machinery in upstream navigation. Zaferani *et al.* implemented the double-cone structure and unraveled butterfly swimming trajectories as a result of potential conservation by spermatozoa while they swim upstream through the narrow regions, where they are exposed to high-magnitude laminar streams ( Zaferani *et al.*, 2019). Tung *et al.* studied the coupling of rheotaxis and surface topography, explicitly focusing on the use of microgrooves. The findings indicate that surface anomalies support the upstream movement of sperm cells under *in vivo* conditions. Quantitatively, a higher subpopulation was observed on the micro-grooved surface compared to the flat surface ( Tung *et al.*, 2014; Tung *et al.*, 2015). Additionally, micro-structures combined with rheotaxis can tether the single sperm cell and facilitate the beating analysis ( You *et al.*, 2019b). Raveshi *et al.* replicated the complex topography of epithelial tissue and encapsulated the sperm cells into different-sized droplets to observe the role of surface curvature in promoting capacitation ( Raveshi *et al*., 2021). Figure 1 encompasses a 2-dimensional illustration of reviewed studies. This depiction elucidates the responses exhibited by swimming spermatozoa in the context of rheotaxis concomitant with microstructure geometries.

The upstream turning, rolling, and spirality of the spermatozoa is arguable, as proposed by Miki and Clapham, suggesting that the turning and rolling of spermatozoa is an outcome of “Cation channel of Sperm (CatSper)” induced [Ca ^++^] signaling cascade ( Miki & Clapham, 2013). However, Schiffer *et al.* and Zhang *et al.* disproved the role of CatSper activation in rheotaxis ( Schiffer *et al.*, 2020; Zhang *et al.*, 2016). Bukatin *et al*. also confirmed the rolling and reorientation of the spermatozoa, which occurs due to the asymmetry in the midpiece. The resultant shear force along the head and flagellum attributes to the reorientation ( Bukatin *et al.*, 2015). These findings substantiate that rheotaxis is a hydrodynamic physical phenomenon, while CatSper-induced motion of the sperm cells may involve additional swimming mechanisms.

### Chemotaxis and thermotaxis

Only capacitated sperm cells exhibit enhanced and more frequent asymmetrical flagellum beating, frequently known as hyperactivated ( Zaferani *et al.*, 2021b). Capacitation is a highly complex phenomenon and is essential for sperm cells to leave the viscous oviductal reservoir, subsequently enabling them to penetrate through cumulus cells and extracellular matrix contiguous to the oocyte. Exclusion of the cholesterol from the sperm membrane, activation of protein kinases, regulation of intracellular pH and membrane potential, increase in intracellular ions level, and production of reactive oxygen species (ROS) are fundamental biomolecular adaptations required for sperm cell capacitation ( Jin & Yang, 2017; Molina *et al.*, 2018). The altered ovarian follicular environment during ovulation has been recommended as the primary driver for sperm capacitation ( Ravaux *et al.*, 2016).

Chemotaxis has been postulated and established as a possible mechanism to explain hyperactivation ( Suarez, 2008). The oocyte secretes specific biochemicals, and the diffusion of released chemicals through a fallopian tube promotes a concentration gradient. The secreted biochemical is called follicular fluid (FF), which contains complex components including heparin and hormones: progesterone, estrogens, atrial natriuretic peptide (ANP), adrenaline, prolactin, vasopressin, oxytocin, calcitonin, and acetylcholine. The binding of chemoattractant molecules stimulates the receptors on flagella and initiates the proliferation of cyclic adenosine monophosphate (cAMP). An increment in cAMP causes the activation of the protein kinase A (PKA). PKA phosphorylates various proteins essential for hyperactivation and fecundation. The cAMP-PKA pathway induces the efflux of K ^+ ^ions through the Slo3 potassium channel and promotes hyperpolarization of the plasma membrane. Subsequentially, the hyperpolarization promotes the opening of calcium channels, which re-settles the hyperpolarization of the membrane—this back-and-forth signaling cascade results in an augmentation of [Ca ^++^] through the flagellar length and altering the beating pattern from symmetrical to asymmetrical ( Molina *et al.*, 2018). Consequently, the chemotaxis is an outcome of the intracellular signaling pathway; the irregularities in associated receptors and in sperm acrosome might potentially alter the fertilization success.

The primary challenges encountered in conducting the chemotaxis study lie in the manipulation of low concentrations of chemoattractant and in establishing a stable gradient with it. Nonetheless, high-degree flow control through microfluidic devices attributes the molecular diffusion, which results in linear/no-linear, precise, and controlled chemical gradients ( Jaberi *et al.*, 2020).

The attainment of linear molecular diffusion can be accomplished with a microfluidic device featuring a central channel and two side branches. Hussain *et al.* correlated individual fertilization with chemotaxis in sea urchins and validated the impact of precise chemoattractant secretion in female fertilization ( Hussain *et al.*, 2016; Hussain *et al.*, 2017). These studies established chemotactic behavior as a marker to assess the fertilizing capability of male and female individuals. However, the presence of fabrication defects and the occurrence of flow-induced pressure imbalances at the contact zone result in uneven mixing ( Deekshith & Jadhav, 2018). Additionally, the excessive width of the principal channel affects the linearity, repeatability, and stability of the gradient.

Eisenbach suggested a qualitative characterization of chemotaxis, which involves the wide-ranging sperm cell trajectories in the chemical gradient field ( Eisenbach, 1999). Indeed, hydrogel-based devices can be potentially exploited for linear gradient generation. The crosslinked network of hydrophilic polymers enables controlled molecular diffusion in such devices. Berendsen *et al.* and Chang *et al.* have studied the trajectories discrepancy within the mouse and sea urchin model utilizing these approaches. The studies signify the relevance of the model selection for sperm chemotaxis. Nonetheless, the repeatability and stability of hydrogel-based devices are questionable, as the molecular absorption of material leads to irregular diffusion in different laboratory environments ( Berendsen *et al.*, 2020; Chang *et al.*, 2013).

**Figure 2.**
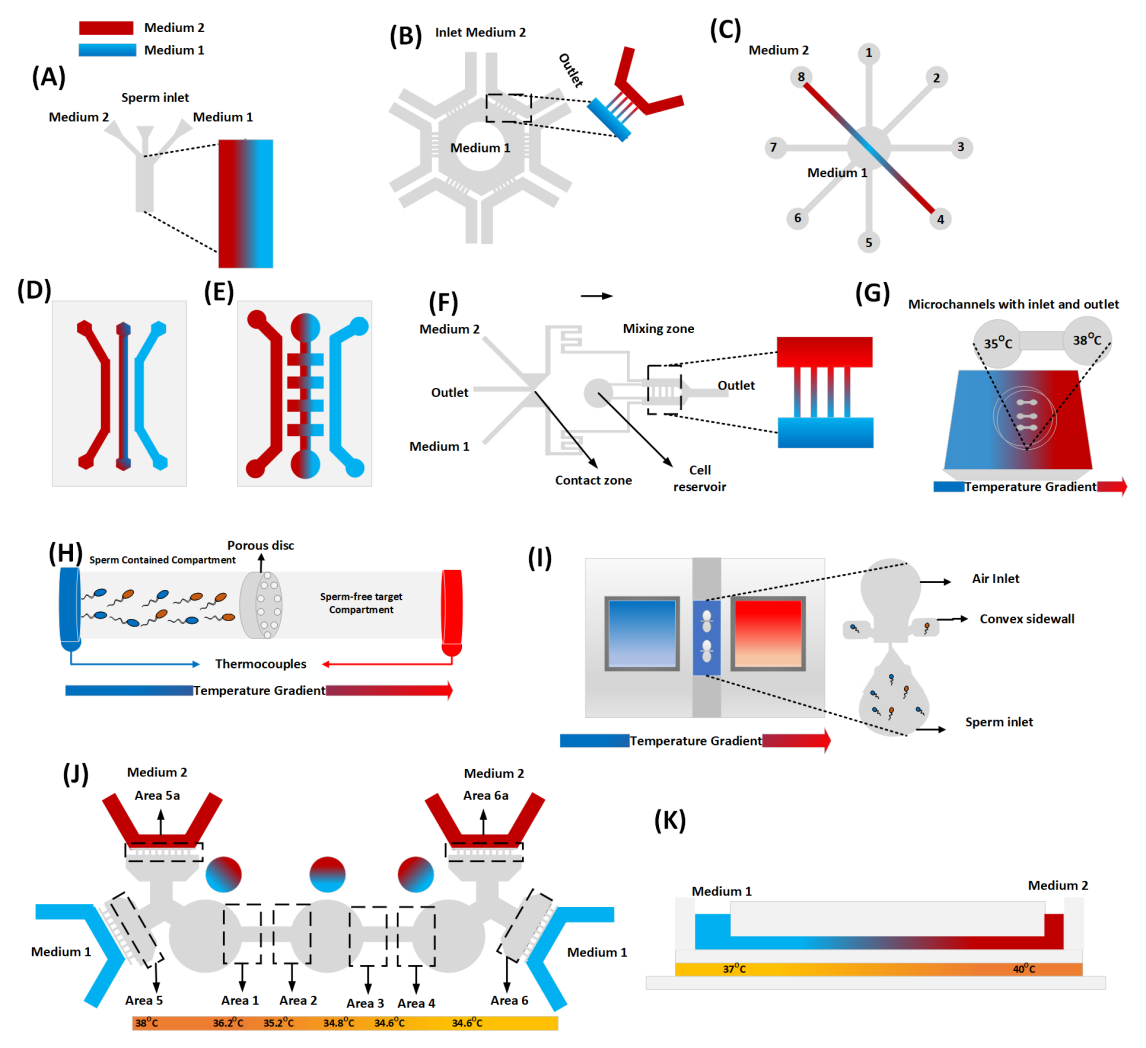
Microfluidic designs for sperm chemotaxis and thermotaxis. Figure ( **A**) reproduces the structure of ( Hussain *et al.*, 2016; Hussain *et al.*, 2017), where a chemical gradient is generated across the centered microchannel (4cm × 1020µm × 99µm) for the sea urchin model. Figure ( **B**) is the adapted design of ( Zhang *et al.*, 2015), where the progesterone gradient occurs in transfer channels (W×H: 5 × 2µm ^2^) that connect the hexagonal pool surrounded and U-shape microchannels (W×H: 700 × 50µm ^2^) connected with the inlet-outlet media. Figure ( **C**) shows the combination of 8 microchannels (10mm×500µm×100µm) that intersect each other at the cell reservoir, with an inlet/outlet positioned at an octagonal configuration, enabling the generation of linear Ach gradient for mouse sperm chemotaxis ( Ko *et al.*, 2012). Figures ( **D**) & ( **E**) illustrate the hydrogel-based chemotaxis devices. The molecular diffusion through the hydrogel facilitates the linear progesterone gradient. Berendsen *et al*. used parallel channels (depth 350 µm) to study boar model (D), while Chang *et al.* used different channel dimensions (W×H: 400 ×167 µm ^2^) to study mouse and sea urchin respectively ( Berendsen *et al.*, 2020; Chang *et al.*, 2013). Figure ( **F**) is the adaptation of ( Bhagwat *et al.*, 2018; Bhagwat *et al.*, 2021), which utilizes the ladder-based design high-fluidic resistance microchannel (1000 × 50 × 50 µm ^3^) to investigate the chemotaxis in the mouse model. Figure ( **G**) reproduces the idea of ( Pérez-Cerezales *et al.*, 2015), where the terminals of a capillary tube are kept at different temperatures (35–38°C) to demonstrate thermotaxis in mouse and human models. Figure ( **H**) recreates the setup of ( Bahat *et al.*, 2012), where a lucite microtube attached with two thermistors at the terminal causes the temperature gradient for the human model (0.5–2 ^O^C). Figure ( **I**) summarizes thermotaxis assays of ( Li *et al.*, 2014) for the human model, where a microfluidic chip like a Dumble-shape is sandwiched between tanks of aluminum alloy (source and sink) filled with glycerol. Figure ( **J**) shows a combined chemotaxis and thermotaxis study ( Yan *et al.*, 2020) in the human model. Three circular chambers facilitate linear and no-linear concentration gradients. Figure ( **K**): presents the temperature and Ach gradient on mouse models ( Ko *et al.*, 2018). The study used simple microchannels (13mm × 500µm × 100µm) integrated with ITO tapes to execute the temperature gradient (~2°C).

Bhagwat *et al.,* reconstituted a ladder-based design with active flow control where one extra outlet was introduced at the contact zone. The additional outlet compensated for the irregular mixing and pressure imbalance at the contact zone. The device was exploited to assess the acetylcholine (Ach)-based chemotactic behaviors in the mouse model and further utilized in discovering novel chemoattractant like N-formyl- _L_-aspartate ( Bhagwat *et al.*, 2018; Bhagwat *et al.*, 2021; Panchal *et al.*, 2022). However, the ladder-based design involves multiple orthogonally positioned channels that can trigger the bubble formation and micro-vertices at the junction corner. Alternatively, the non-linear gradient can be utilized, like Zhang *et al.* established a non-linear concentration ramp for the progesterone characterization ( Zhang *et al.*, 2015). Figures 2A to 2F represent the microfluidic-based approaches that have been exploited for the generation of chemical gradients.

The molecular mechanism underlying hyperactivation remains elusive. For instance, chemotaxis predominantly occurs in relatively small regions near the oocyte, whereas hyperactivation commences far from the oocyte location. Thermotaxis is postulated to be the long-distance directing mechanism in the follicular tube ( Bahat *et al.*, 2003). Sperm cells comprise G-proteins receptors, including opsins and transient receptor potential (TRP) channels, which are fundamentally involved in the regulation of phototransduction. TRP channels and opsin are involved in increasing intracellular calcium levels and guiding the hyperactivated swimming of sperm cells through the oviductal funnel ( Pérez-Cerezales *et al.*, 2015; Xiao *et al.*, 2022). Figure 2G to 2K demonstrates the microfluidic designs employed in thermotaxis studies.

It is not so straightforward to reproduce the *in vivo* conditions related to thermotaxis, as the magnitude of the gradient is very small (~2°C), and the range of the gradient is not comprehended adequately ( Bahat *et al.*, 2003; Bahat & Eisenbach, 2006). Bahat *et al*. developed a biological assay to characterize thermotactic sperm response. The study reported the kinematics of progressive sperm cells along temperature gradients of ~2°C, noting that the instantaneous velocity and motility of sperm cells deteriorate above 40°C ( Bahat *et al.*, 2012). Li *et al*., developed an assay to isolate thermotactic sperm cells using a microfluidic device placed between two heat sources that exploit glycerol for higher gradient stability ( Li *et al.*, 2014).

Pérez-Cerezales *et al*., established the clinical relevance of thermotaxis. The developed assay comprises a capillary set at ~3°C temperature gradient. The temperature-based migration was recorded, and trajectory analysis of sperm cells unveiled that thermotactic cells exhibit higher progressivity in human model, whereas progressivity diminishes in mice. Additionally, the immunocytochemical assay revealed the heterogeneity in rhodopsin location in non-selected sperm cells, affecting thermo-sensing capabilities. The additional biological validation included *in vitro* development of the rat embryos utilizing thermotaxis and conventional swim-up assays. The morphological analysis of the morula, blastocyst, and cell division stage confirmed the extended hatching of zygotes and validated the significance of thermotaxis ( Pérez-Cerezales *et al.*, 2015). 

Reviewed studies have substantiated the efficacy of microfluidics-based devices in elucidating the complexities related to with *in-vivo* interactions between sperm cells and the female reproductive tract. The outcomes can be potentially translated into developing efficient sperm separation devices. However, the capability to characterize the physiological parameters like pH, temperature, and concentration of chemoattractant(s) for the development of reproductive medicine has not been sufficiently exploited for the human model. Few studies investigated the optimum physiological environment for the bovine model and analyzed the impact of Kisspeptin-10 on the Ram model ( Abdel-Ghani *et al.*, 2020; El-sherry *et al.*, 2017; EL-sherry *et al.*, 2020). Nonetheless, sperm physiology differs even within mammal specimens ( Molina *et al.*, 2018; Romero-Aguirregomezcorta *et al.*, 2018), and the lack of studies on the human model is evidential.

Indeed, microfluidic-based devices offer promising potential for concurrent biomimicking of rheotaxis, thermotaxis, and chemotaxis. A few studies have demonstrated the possibilities and analyzed the swimming migration and tail beating in bi-gradient space (temperature and chemical) ( Ko *et al.*, 2018; de Wagenaar *et al.*, 2016b; Yan *et al.*, 2021). Nonetheless, translating these studies for quality sperm selection requires an improved representation that involves biological and clinical validations.

Chemotaxis and thermotaxis have been correlated with the fertilization capability of spermatozoa. However, there has been no significant advancement in studying human sperm chemotaxis. For example, the high degree of control and the geometric versatility of microfluidics should be better exploited to improve the characterization of the biochemical composition of FF and its interaction with spermatozoa. Indeed, progesterone is the only widely tested chemoattractant in the human model, but its role as a chemoattractant is debatable, as the absence of progesterone from FF did not diminish the chemotactic behavior but altered the hyperactivation of spermatozoa ( Jaiswal *et al.*, 1999). Few studies identify crucial signaling proteins, such as epidermal growth factor (EGF), brain-derived neurotrophic factor (BDNF), vascular endothelial growth factor (VEGF), insulin-like growth factor (IGF1), and leptin, which plays a vital role in oocyte maturation and might contribute to the spermatozoa signal transduction pathway ( Lakshminarasimha *et al.*, 2022; Li *et al.*, 2020; Prochazka *et al.*, 2017). However, there is a lack of studies establishing mentioned proteins as chemoattractant(s). Nonetheless, for sperm cells, the Ach triggers EGF receptor phosphorylation, leading to increased calcium ions during the acrosome reaction in the mouse model ( Jaldety *et al.*, 2012; Ko *et al.*, 2012). As of now, there is a dearth of empirical evidence supporting the existence of such mechanisms in the human model.

## Microfluidics-based sperm selection

The motility of the spermatozoa is likely the most exploited marker for microfluidics-based sperm cell separation. A microfluidic device containing a straight microchannel that bifurcates into sub-microchannels near the inlets and outlets was employed, characterized, and optimized for the quality subpopulation ( Matsuura *et al.*, 2012; Shirota *et al.*, 2016). Chen *et al*., delivered a microfluidic device inspired by the conventional swim-up method; however, the study did not report analysis for quality parameters against the conventional centrifugation-based methods ( Chen *et al.*, 2013b). Riordon *et al*., delivered the DNA-intact subpopulation by separating the plane swimmer. Nonetheless, the recovery of the subpopulation was very low ( Riordon *et al.*, 2019). Eamer *et al*., and Nosrati *et al*. exploited the biophysics of sperm-wall interaction and delivered rapid qualitative selection ( Eamer *et al.*, 2016; Nosrati *et al.*, 2014). Subsequently, Vasilescu *et al*., modified the device and introduced microgrooves and a magnetic beads chamber to increase the concentration and quality of the subpopulation collection ( Vasilescu *et al.*, 2023).

Moon *et al*., invented a simple microchannel for sperm separation ( Moon *et al.*, 2009), later adopted by Tasogulu *et al.,* who reported the significance of assay duration ( Tasoglu *et al.*, 2013a). The device, known as Fertile®, has been commercialized by Zymot Fertility© and Koek biotechnology©. Several studies claim that Fertile® can effectively separate high DNA intact cells ( Anbari *et al.*, 2021; Gode *et al.*, 2019; Gonzalez-Castro & Carnevale, 2019; Quinn *et al.*, 2018), although clinical studies have shown no improvement in fertilization success rates ( Yalcinkaya Kalyan *et al.*, 2019). Chinnasamay *et al*., modified the device by introducing cylindrical obstacles for enhanced quality recovery ( Chinnasamy *et al.*, 2018).

**Figure 3.**
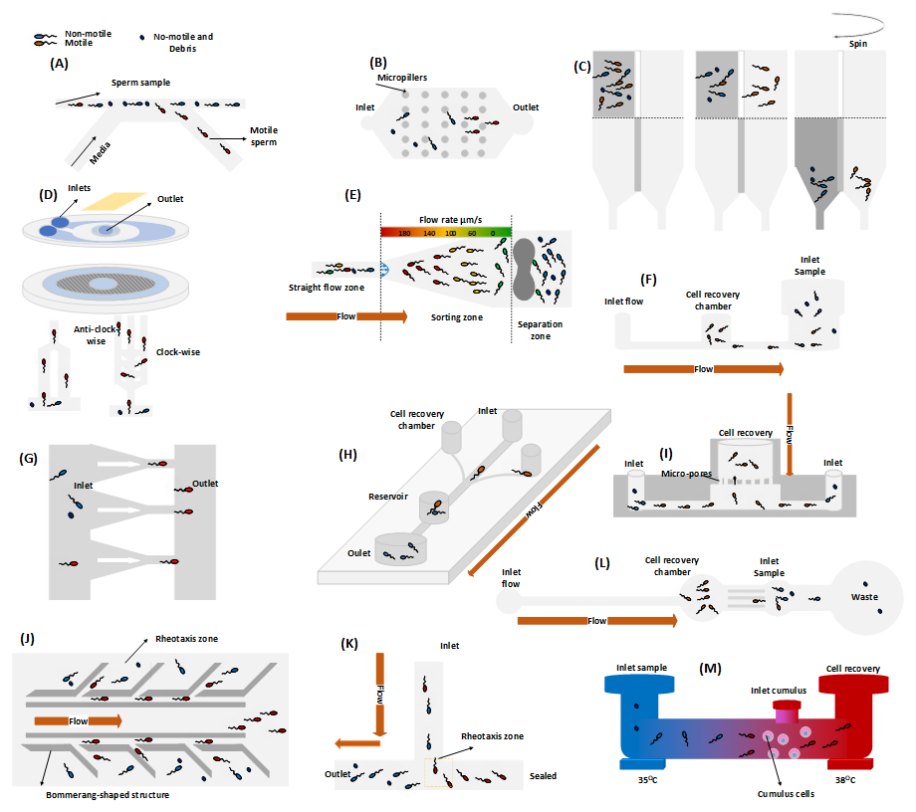
Quality subpopulation extraction utilizing microfluidic models. The device descriptions and the corresponding findings are in supplementary table 1. Figure ( **A**) illustrates the device concept utilized in the studies conducted by ( Matsuura *et al.*, 2012; Shirota *et al.*, 2016). Figure ( **B**) depicts the device (without cylindrical obstacles), initially introduced by ( Moon *et al.*, 2009) and further tested on a human model by ( Tasoglu *et al.*, 2013b; Zhang *et al.*, 2011). The device, commercially available as Fertile®, was subsequently modified by ( Chinnasamy *et al.*, 2018) by incorporating micro-obstacles to improve its quality metrics. Figure ( **C**) demonstrates the design and protocol developed by ( Chen *et al.*, 2013b), which is inspired by the conventional swim-up method for quality sperm cell separation. Figure ( **D**) shows the radial fluidic design invented by ( Nosrati *et al.*, 2014) for rapid, high-DNA intact cell separation. ( Eamer *et al.*, 2016) modified the device to separate clockwise and anti-clockwise swimmers, while ( Vasilescu *et al.*, 2023) introduced microgrooves and magnetic beads chamber to enhance the separation efficacy of the device. Figure ( **E**) is adapted from ( Wu *et al.*, 2017) and showcases a dumbbell-like structure that effectively obstructs debris and non-motile sperm cells, thereby promoting rheotaxis-based cell separation. Figure ( **F**) represents the cell separation concept of ( Rappa *et al.*, 2018). The active flow driven system promotes rheotaxis, while a chamber positioned between flow inlet and outlet (sample inlet) facilitates the recovery of cells. Figure ( **G**) demonstrates the device of ( Riordon *et al.*, 2019) for planer-swimmer sorting. Figure ( **H**) shows the rheotaxis based cell sorting by ( Romero-Aguirregomezcorta *et al.*, 2021). Figure ( **I**) replicates passive-flow driven device based on rheotaxis and a membrane for quality cell separation ( Ataei *et al.*, 2021). Figure ( **J**) illustrates the design proposed by ( Zeaei *et al.*, 2023), where boomerang-shaped structure is employed to promotes rheotaxis for the separation of quality sperm cells. Figure ( **K**) showcases the T-shaped design, where sample insertion generates flow and promotes rheotaxis for the quality sperm collection ( Mane *et al.*, 2022). Figure ( **L**) represents an active flow-driven device for the separation of the cells ( Sharma *et al.*, 2022). Figure ( **M**) represents a device for the separation of sperm cells based on chemotaxis and thermotaxis in the human model ( Doostabadi *et al.*, 2022).

Motility alone does not exclusively determine natural fertilization. Despite the unresolved physiological mechanisms of sperm cells in human models, previously discussed microfluidic-based approaches can be potentially exploited to deliver quality sperm separation. The rheotaxis-based devices combined with biomimicking of reproductive tract anatomy have emerged as a prominent approach ( Ataei *et al.*, 2021; Rappa *et al.*, 2018; Romero-Aguirregomezcorta *et al.*, 2021; Sharma *et al.*, 2022; Wu *et al.*, 2017; Zeaei *et al.*, 2023). A detailed description, findings, advantages, and disadvantages can be found in Table 1. Additionally, Figure 3 elucidates the design and protocol conceptions outlined in the cited scientific journals that were employed for quality sperm selection. It is evident from the table that reviewed technology does not fully address the unmet needs of the reproductive clinicians, primarily the lack of separated concentrations and the laborious protocol. Additionally, the lack of extensive research on chemotaxis and thermotaxis has significantly affected the employability of microfluidics approaches for separating capacitated cells. To date, ( Doostabadi *et al.*, 2022) is the only study that has demonstrated improved quality metrics by utilizing chemotaxis and thermotaxis to separate human sperm cells. However, it is worth noting that despite the improved quality, the separated cells still exhibit significant DNA fragmentation ( Figure 3). 

**Table 1.** Summary of reviewed microfluidic devices for quality sperm selection employed in the human model.

Journal publications	Method		Material	Device description	Advantages/ outcomes	Disadvantages
	Mechanism	Flow Control				
( Matsuura *et al.*, 2012)	Motility	No-flow	COP ^1^	The symmetric microfluidic device contains a straight microchannel that bifurcates into two sub- microchannels at both terminals ( Cho *et al.*, 2003). The cells with higher motility showed proficiency in swimming across the main channel. On the contrary, debris and non-motile cells flow away with the streams. Channels with different configurations (H × W: 0.3mm × 0.5mm: 0.1mm × 0.6mm) were tested to improve the efficacy of the device.	-Recovered cells with ~90%. motility -Validated DNA integrity in a previous study ( Schulte *et al.*, 2007)	-Low recovery subpopulation (0.2-0.3%)
( Tasoglu *et al.*, 2013a) ( Zhang *et al.*, 2011)	Motility	No-flow	PMMA ^2^	The “Space Constrained Microfluidic Sorting (SCMS)” was invented by ( Moon *et al.*, 2009) and was adapted to optimize assay length. The microchip comprising 50 µm deep microchannels with various lengths was implemented to characterize sperm kinematics. More extended channels yield less subpopulation of sperm cells with increased dynamics. Chip was also tested for the human model.	-Significance of the length of the assay -Recovered yield of ~1.6×10 ^6^ ( Zhang *et al.*, 2011). -Clinical validation -Commercialized as Fertile ^®^ Chip	-Low % recovery against conventional -Not recommended for cryopreserved sample -Unhandy protocol
( Chen *et al.*, 2013b)	Motility	No-flow	PDMS ^3^	The study is inspired by the conventional swim-up method. The chip encompasses two miniaturized compartments with small pellets. The left-side compartment holds the semen sample, and the right contains the sperm preparation medium. The Brownian motion of motile cells encourages the swimming of the subpopulation (~50% of motile sperm cells) towards the compartment filled with buffer. Subsequently, density centrifugation assists in the sedimentation of sperm concentrations into pellets.	- Semen analysis validated against microscopic Makler chamber method (<5%).	-Centrifugation- based separation -Lack of DNA fragmentation test
( Nosrati *et al.*, 2014)	Motility	No-flow	PDMS	The radial shape design includes two layers: the bottom-part hosts 500 microchannels (100µm × 75µm) and a top-layer structure containing the inlet and outlet connected with the round channel. The coupling of both fluidic layers accomplished the fluidic circuits. The channel lengths were characterized to optimize the device’s efficacy.	- Validation against conventional methods -Subpopulation vitality (~98%) and concentration reported (~0.6%) -Chromatin test- based DNA integrity validation (~98%).	-Low subpopulation recovery in comparison to conventional methods
( Shirota *et al.*, 2016)	Motility	No-flow	COP	Device design and functioning are explained in ( Matsuura *et al.*, 2012).	-Clinical validation against conventional methods -Obtained subpopulation motility >95% with 0.8% DNA fragmentation.	-Low subpopulation recovery in comparison to the centrifugal method (~3.6 × 10 ^6^) -Not recommended for IUI ^4^
( Eamer *et al.*, 2016)	Motility	No-flow	PDMS	As explained by ( Nosrati *et al*., 2014), the radial-chip concept was re- implemented for sperm selection. The chip has been modified, allowing the subpopulation recovery of clockwise, anti- clockwise, and straight micro- swimmers. The radial-shape design contains 52 channels (7.0cm×100µm×60µm). Additionally, three chips comprising the microchannels with side branches at different angles (45°,90°, and 135°) were demonstrated to optimize the angle of attack for boundary swimmers.	-High-yield (~4million/ml) ->80% vitality -DNA fragmentation validation (~98% DNA intact cells) -Junction angles optimization (45°)	-Lack of validation against currently practiced methods
( Wu *et al.*, 2017)	Rheotaxis	Passive	PDMS	The study considered the physiology of the female reproductive tract while sperm cells swim from the vagina toward the uterus, perceiving a downstream flow of the vaginal discharge. A diffusive structure microchannel (L×H: 1cm ×100µm) and hydrostatic pressure-driven flow were combined to mimic the rheotaxis.	-Clinical Validation -Proved efficacy against swim-up and DGC methods -Reported highly motile sperm cells separation in infertile patients (~22%) -Subpopulation recovery ~2×10 ^6^ cells/ml with ~90% motility.	-Lack of DNA integrity validation - Low recovery of the separated cells
( Chinnasamy *et al.*, 2018)	Motility	No-flow	PDMS	A simple periodic micro-cylindrical (D: 10µm, H:50µm) obstacle was employed in simple microchips as explained n ( Tasoglu *et al.*, 2013a). The recovered subpopulation was validated against the plane channels and swim-up method.	-Quality validation against swim-up -Morphology validation (>55%) -The chromatin test validates a higher percentage of mature sperm (>43.5%) -~5% DNA fragmentation	~Low recovery efficiency (<2%)
( Rappa *et al*., 2018)	Rheotaxis	Active	PMMA	A simple microchannel of dimension (22.4mm × 4mm × 76µm) connected to the inlet, reservoir, and a midway collection chamber. Rheotaxis experimentation was conducted at 2,4,5,6,8 and 10µl/min. Hyaluronic and morphological assays did not indicate a significant difference with executed cell separation assays, contrary to ( Romero-Aguirregomezcorta *et al*., 2021).	-Quantitative analysis of recovered subpopulation (<20% recovered cells with ~80% motility).	-Lack of validation against practiced methods -Missing DNA fragmentation test -Comparatively less motility compared to the Fertile ^®^
( Riordon *et al.*, 2019)	Motility	No-flow	Glass	Two parallel zigzag microchannels (30cm long) were designed for cell separation. The channels were connected through 3250 channels (L×H: 400µm × 2µm). The channel height is in order of cell head thickness, resulting in only the plane swimming of spermatozoa. The study found that the sperm cells with planner swimming carry better DNA integrity and validated against the density gradient and swim-up methods.	-Clinical validation against swim-up and DGC ^5^. -Recovered cell DNA integrity is ~90%	-Low subpopulation recovery ~0.1% -Discarded helical swimming
( Romero-Aguirregomezcorta *et al.*, 2021)	Rheotaxis	Active	PMMA	A microchannel of dimension (10mm × 300 µm × 100 µm) was exposed to the inlet-outlet of the channel. The flow was induced through a syringe pump (30µm/s), and 40µl of subpopulation was accumulated midway through the channel. The motility, DNA integrity, and morphology of the separated specimen were validated against conventional methods. The rheotaxis-based selection improves the ICSI ^6^ outcomes.	-Recovered 100% motility and >80% viability -DNA fragmentation <5% >50% normal morphology More than 20% higher fetal development	- Processing of low semen volume -Unhandy protocol -Not convenient for other ART methods like IUI and IVF ^7^.
( Ataei *et al.*, 2021)	Rheotaxis	Passive	PMMA	A passive flow device comprises a microchannel with two inlets at the terminal and an outlet in the middle. The outlet of the channel facilitates the hydrostatic pressure difference for flow and holds a membrane with holes for separation. Morphology and DNA fragmentation was validated, and a flow velocity of 25µl/min was reported for the optimized cell separation.	-Isolation efficacy ~30% -Morphological, DNA, and CASA ^8^ validation ->45% normal morphology -~5% DNA fragmentation	- Lack of validation against practiced methods -45 minutes long assays
( Doostabadi *et al.*, 2022)	Chemotaxis/ Thermotaxis	No-flow	3D- printing resin	A simple microtube with a 2mm diameter connects two cylindrical chambers (H×D: 9mm × 5mm). The length of the microtube is 2cm, including a hole near one chamber for the insertion of cumulus cells. Both chambers are kept at different temperatures, respectively 35°C and 38°C, to provoke thermotaxis and chemotaxis-based sperm migration.	- Validation against direct swim-up - High concentration separation (>30%) -Better DNA integrity against swim-up -toxicity validation	-Separated cells comprise >10% fragmented cells -1hrs long protocol -Instability in temperature gradient
( Sharma *et al.*, 2022)	Rheotaxis	Active	PDMS	A device with a 96 µm height comprises a 20 mm long microchannel (W: 300 µm) that connects the collection chamber and the flow inlet unit. The collection chamber is connected to the sample inlet via multiple sub-microchannels (W×L: 300 µm × 5 mm) integrated into a 2.4 mm channel width. The same channel is extended without sub- microchannels to connect to the waste chamber.	->95% motility of the separated cell -~1.5% separation efficacy -DNA fragmentation validation (<2.5%)	- Lack of validation against conventional practice -Low- concentration recovery -Missing protocol timeline
( Mane *et al.*, 2022)	Rheotaxis	Passive	PDMS	A pre-filled T-shaped microfluidic device (W×H: 200 µm ×50 µm), sealed at one side of the horizontal channel, is used to utilize a 40 µl semen sample and generate gravity-assisted diffuse flow. Rheotaxis occurs adjacent to the junction due to the inlet-outlet pressure imbalance, causing motile cells to migrate toward the sealed channel.	-Inexpensive protocol - Nitro-blue tetrazolium (NBT) validation -~100% motile cells recovery	-2hrs long protocol -The separated concentration was not mentioned -Lack of validation against conventional practices
( Zeaei *et al.*, 2023)	Rheotaxis	Active	PDMS	The device comprises a central channel connected to two parallel channels (500 µm) with boomerang-shaped structures. The optimal shear rates (3.5-6 s-1) were achieved in the contact region (rheotaxis zone). Different depths of the designs were tested, including 500 µm, 200 µm, and 100 µm.	~160 000 motile cells in 20 min >95% motility of separated cells -ICSI favorable	-Meager sample collection for normal mother sample -Not recommended for IUI and IVF -More than >3% of cells are fragmented
( Vasilescu *et al.*, 2023)	Motility	No-flow	3D- printing resin	The device, inspired by ( Nosrati *et al.*, 2014), underwent modifications to improve its efficacy. Multiple microgrooves were introduced to the connection wall in the z-direction. Additionally, a magnetic microbeads chamber was created to trap phosphatidylserine- positive sperm.	->5% of separation recovery -~100% vitality -~100% motility -very low DNA fragmentation (<1.4%) -Short-duration protocol	-Not appropriate for IUI

^1^Cyclic olefin copolymer (COP) ^2^Poly(methyl methacrylate) (PMMA) ^3^Polydimethylsiloxane (PDMS) Note: To explain microfluidic dimensions height, width, length, and diameter denoted with H, W, L, and D respectively ^4^Intrauterine insemination (IUI) ^5^Density gradient centrifugation (DGC) ^6^Intracytoplasmic sperm injection (ICSI) ^7^Invitro fertilization (IVF) ^8^Computer-assisted semen analysis (CASA)

## Recommended commercialization pathway

As established in this review, microfluidic-based sperm selection approaches have shown the potential to facilitate high-quality sperm cells. However, the viability of microfluidics in life science applications is debatable, as the technology has not lived up to the initial hype during its early developmental phase (1990–2009) ( Mukhopadhyay, 2009). Materials, modeling, and machinery play a significant role in upsurging the technology's readiness level (TRL). In the aspects of microfluidic application for sperm selection, the rate of technology translation is still meager. The authors can allude to three possible reasons: 1) the developed technologies are still in the early stage of development; 2) there is a lack of communication between the end-users and the researchers; and 3) there is a potential lack of interest by the industry to change current methods unless costs, efficacy, and usability are maintained or improved.

An innate communication disparity among academia, industry, and the end-user is the most critical impediment towards commercialization readiness of the developed technologies. For example, most microfluidic engineers practice rapid prototyping using Polydimethylsiloxane (PDMS). Our review analysis indicated that approximately 70% of researchers opt for PDMS and hydrogel-based prototyping. However, the materials are not very well-suited for biological applications. Fluidic chips made of these materials comprise several shortcomings, including their tendency to absorb small hydrophobic molecules, release tiny polymeric fragments, and are relatively expensive for large-scale production (Mukhopadhyay, 2007). Furthermore, the conventional UV-lithography for the SU-8 mold fabrication is associated with repeatability issues, high maintenance requirements, a sophisticated environment, and a specialized workforce.

Hence, it is evident that researchers should consider adopting a mitigation plan or show the possibility of using alternative materials to enhance the readiness level of prototyping. High-resolution biocompatible resin-based 3D printing can serve as a viable alternative for prototyping fluidic devices, offering improved repeatability and scalability for device fabrication. Nevertheless, 3D-printing-based prototyping faces limitations regarding the printing of microchannels dimensions printing and tolerances. Therefore, these prototypes should incorporate feasible printing dimensions and further promote user-friendly protocol. Subsequently, thermoplastics-based devices are recommended for industrialization, especially cyclic olefin copolymer (COC/COP) and polymethyl methacrylate (PMMA). Both polymers have FDA approval for medical applications; moreover, lower biomolecular absorption, ease of fabrication, and cost-effectiveness with industry-ready methods and sustainability of these polymers encourage their utilization in large-scale fabrication of microfluidic chips.

In the context of clinical implementation of microfluidic-based approaches, low throughput appears as a principal hurdle. The associated scientific community has undoubtedly discovered exceptionally engineered microfluidic approaches for quality sperm selection. However, clinical screening requires a statistical analysis over a wide range of human semen samples. The limited recovery of sperm cell concentration poses a constraint on reproductive laboratories, hindering their ability to execute reproductive procedures like IUI and IVF. As a result, reviewed microfluidic-based methods lack clinical validation, where the biological validation associated with quality sperm selection has not been correlated with the pregnancy rate and the quality of embryos. Additionally, centrifugation-based protocols offer rapid processing of multiple samples, while microfluidics-based methods require multiplexing for single-sample processing, potentially resulting in a less user-friendly protocol. Despite the better-quality outcomes offered by microfluidics-based approaches, they have not gained much attention due to their long and tedious protocols. The emphasis on developing proof-of-concept without analyzing the unmet needs of end-users has influenced the translation of technology.

The commercial execution of medical devices —especially for the class 1-3 category— necessitates regulatory approvals. The ascending numeric assignment (1 to 3) correlates with a proportional escalation in the associated risk level with the implication of the device ( Marešová *et al.*, 2020a). In the context of the assisted reproduction, where the disposable devices correspond to the class 2a to 3, which incur substantial costs and labor efforts ( Heinemann, 2021; Marešová *et al.*, 2020b). Additionally, the cost of production in ISO environments and logistics accumulate the expenses associated with the developed medical disposable. Such significant investments cannot be solely supported by academia. On the contrary, end-users have low willingness-to-pay (WTP), as the current conventional methods, including DGC and swim-up, offer low processing costs and high throughput. Hence, the commercial translation of such technology (high investment demand with low WTP) is only feasible if the involved industry or academia is supported by investors/business angels/venture capitalists.

## Ethics and consent

Ethics approval and consent were not required.

## Data Availability

No data are associated with this article
