# Supplementary material for: Lab-on-chip (LoC) application for quality sperm selection: An undelivered promise?
Source: Open Res Eur. 2023 Oct 30;3:188. [Version 1] doi: 10.12688/openreseurope.16671.1 (PMC11031645; doi:10.12688/openreseurope.16671.1)
Supplement: Peer review history

# Reviewer report: Reviewer response for version 1

**DOI:** 10.21956/openreseurope.18003.r36874

**Published:** 2024-04-19

**Authors:** Abhijit Majumdar

**Affiliations:** 1 Department of Chemical Engineering, Indian Institute of Technology Bombay, Mumbai, Maharashtra, India

The review article titled "Lab-on-chip (LoC) application for quality sperm selection: An undelivered promise?" by Shukla *et al.* is a nicely written article covering adequately the recent progresses in microfluidic based sperm sorting techniques. I have only two comments to make:

1. This article differentiates "Active" and "passive" based on the mode of fluid flow. However, in a more commonly adopted classification, "active" sperm sorting is based on the flow (irrespective of the technique used to flow the fluid). In active mode, sperms are sorted based on their rheological dynamics (ability to swim). In "passive" mode, they are sorted based on their physical properties such as shape and size. In my opinion the classification made in Huang *et al. *(2023 ^1^) is more accurate. 
2. The article will be more useful if the authors discuss about the sperm and fertilisation physiology with greater details. Such introduction will be very useful for the uninitiated readers, in my opinion.

Is the review written in accessible language?

Yes

Are all factual statements correct and adequately supported by citations?

Yes

Are the conclusions drawn appropriate in the context of the current research literature?

Yes

Is the topic of the review discussed comprehensively in the context of the current literature?

Yes

Reviewer Expertise:

Microfluidics, organ on chip, mechanobiology

I confirm that I have read this submission and believe that I have an appropriate level of expertise to confirm that it is of an acceptable scientific standard, however I have significant reservations, as outlined above.

## References

[1] : Emerging microfluidic technologies for sperm sorting. *Engineered Regeneration* .2023;4(2) : 10.1016/j.engreg.2023.02.001 161-169 10.1016/j.engreg.2023.02.001

# Reviewer report: Reviewer response for version 1

**DOI:** 10.21956/openreseurope.18003.r39111

**Published:** 2024-04-19

**Authors:** Natan T Shaked

**Affiliations:** 1 Tel Aviv University, Tel Aviv, Israel

The paper presents a review on lab-on-chip technologies for sperm evaluation. My remarks appear below:

 The paper is mostly focused on scientific contributions to the field.  But there is a need to add the technologies that are available for purchase in the market and a strengths and weaknesses comparison. 

 The paper misses some technologies that uses microfluidics for sperm morphological analysis, such as Eravuchira *et al.* (2018 ^1^). 

 Is there any technology that allows single sperm selection in the same ICSI dish of the sperm to egg injection?

Is the review written in accessible language?

Yes

Are all factual statements correct and adequately supported by citations?

Partly

Are the conclusions drawn appropriate in the context of the current research literature?

Partly

Is the topic of the review discussed comprehensively in the context of the current literature?

Partly

Reviewer Expertise:

Sperm imaging and selection. Circulating tumor cells, Interferometry

I confirm that I have read this submission and believe that I have an appropriate level of expertise to confirm that it is of an acceptable scientific standard, however I have significant reservations, as outlined above.

## References

[1] : Individual sperm selection by microfluidics integrated with interferometric phase microscopy. *Methods* .2018;136: 10.1016/j.ymeth.2017.09.009 152-159 10.1016/j.ymeth.2017.09.009 28958952

# Reviewer report: Reviewer response for version 1

**DOI:** 10.21956/openreseurope.18003.r38062

**Published:** 2024-04-19

**Authors:** David Martin-Hidalgo

**Affiliations:** 1 University of Extremadura, Caceres, Spain

In this review by Shukla *et al.*, the authors make a great effort to summarize the Lab-on-a-Chip (LoC) application for quality sperm selection, with a special focus on rheotaxis, thermotaxis, and chemotaxis. The review is well-written and easy to follow. The figures are very illustrative and contribute to understanding the microfluidic models. However, the flow arrows in Figure 1 are hard to see in terms of direction, and I recommend increasing the size of the arrows.

 On page 3, you stated, 'We have created a reference database through PubMed® and reviewed the last ten years of research articles attributed to keywords, including rheotaxis, chemotaxis, and thermotaxis.' Typically, in a review, you should specify the number of studies included, as well as those excluded and the reasons for exclusion.

 The table is excellent; the authors summarized the main results of many manuscripts. However, I feel that there is a lack of discussion in the text regarding the pros and, more importantly, the cons of using the LoC for sperm. I highly recommend that the authors expand this aspect in a new subsection or include it in the 'Recommended Commercialization Pathway' section of their review.

Is the review written in accessible language?

Yes

Are all factual statements correct and adequately supported by citations?

Yes

Are the conclusions drawn appropriate in the context of the current research literature?

Yes

Is the topic of the review discussed comprehensively in the context of the current literature?

Yes

Reviewer Expertise:

reproduction, sperm quality, sperm capacitation, intracelullar pathways.

I confirm that I have read this submission and believe that I have an appropriate level of expertise to confirm that it is of an acceptable scientific standard.
